# Genome-wide detection of copy number variations using high-density SNP genotyping platforms in Holsteins

**DOI:** 10.1186/1471-2164-14-131

**Published:** 2013-02-27

**Authors:** Li Jiang, Jicai Jiang, Jie Yang, Xuan Liu, Jiying Wang, Haifei Wang, Xiangdong Ding, Jianfeng Liu, Qin Zhang

**Affiliations:** 1 Key Laboratory of Animal Genetics, Breeding and Reproduction, Ministry of Agriculture, College of Animal Science and Technology, China Agricultural University, Beijing, 100193, P. R. China; 2 Shandong Provincial Key Laboratory of Animal Disease Control and Breeding, Institute of Animal Science and Veterinary Medicine, Shandong Academy of Agricultural Sciences, Jinan, 250100, China

**Keywords:** Copy number variations, Cattle, BovineHD beadChip, Genome variation, Quantitative real time PCR

## Abstract

**Background:**

Copy number variations (CNVs) are widespread in the human or animal genome and are a significant source of genetic variation, which has been demonstrated to play an important role in phenotypic diversity. Advances in technology have allowed for identification of a large number of CNVs in cattle. Comprehensive explore novel CNVs in the bovine genome would provide valuable information for functional analyses of genome structural variation and facilitating follow-up association studies between complex traits and genetic variants.

**Results:**

In this study, we performed a genome-wide CNV detection based on high-density SNP genotyping data of 96 Chinese Holstein cattle. A total of 367 CNV regions (CNVRs) across the genome were identified, which cover 42.74Mb of the cattle genome and correspond to 1.61% of the genome sequence. The length of the CNVRs on autosomes range from 10.76 to 2,806.42 Kb with an average of 96.23 Kb. 218 out of these CNVRs contain 610 annotated genes, which possess a wide spectrum of molecular functions. To confirm these findings, quantitative PCR (qPCR) was performed for 17 CNVRs and 13(76.5%) of them were successfully validated.

**Conclusions:**

Our study demonstrates the high density SNP array can significantly improve the accuracy and sensitivity of CNV calling. Integration of different platforms can enhance the detection of genomic structure variants. Our results provide a significant replenishment for the high resolution map of copy number variation in the bovine genome and valuable information for investigation of genomic structural variation underlying traits of interest in cattle.

## Background

Recent studies have discovered an abundance of copy number variations (CNVs) in human and domestic animal genomes [1-9]. CNV is defined as a variable copy number of DNA segments ranging from 50bp to several megabases (Mb) compared with a reference genome [3]. The initial study from the analysis of the human genome indicated that single-nucleotide polymorphisms (SNPs) are the most important source of genome sequence diversity and the main contributors to phenotypic variation, environmental response and disease susceptibility [10]. However, the first two genome-wide scans of CNVs in the human genome, which are considered as landmark of CNV studies, have showed that CNVs distribute ubiquitously in the genome [2,11] and are important source of genetic variance [12]. Since then, thousands of novel CNVs were detected in the human genome [13-16]. So far, there are 179,450 CNVs identified in the human genome (Database of genomic variants, DGV: http://dgvbeta.tcag.ca/dgv/app/home?ref=NCBI36/hg18) which cover more than 53% human genome. Besides in human, CNVs have been also identified in many other species, including mouse [17-19], fruit fly [20], dog [9], pig [6,21,22] and cattle [4,5,8,23-26].

It has been revealed that although CNVs account for a smaller proportion of all variations comparing with SNPs, they involve larger genome region of all variant bases [27] and can potentially influence phenotypes or lead to diseases by employing a wide variety of mechanisms, such as changing gene dosage, disrupting genes structure [28,29] and altering gene expression by exposing recessive alleles or indirectly through disturbing the regulation regions of genes [30]. Multiple studies in human have identified that CNVs contribute to phenotypic diversity and complex diseases such as developmental delay, systemic lupus erythematosus, autism and neuroblastoma [31-36]. Phenotype variations caused by CNVs were also observed in domestic animals. For instance, the Pea-comb phenotype in chicken is caused by the duplication of the first intron of the *Sox5* gene [37]. The white coat phenotype in pigs is caused by the copy number variation in the *KIT* gene [38] and the white and grey coat colour in sheep is caused by the copy number variation in the *ASIP* gene [39] . A duplication encompassing the *FGF3*, *FGF4*, *FGF19* and *ORAOV1* genes lead to dorsal hair ridge and susceptibility to dermoid sinus in dogs [40]. It was also reported that CNVs may be associated with many diseases and developmental abnormalities in domestic animals, such as cone-rod dystrophy 3 [41] and startle disease in dogs [42], osteopetrosis and abortions and stillbirths in cattle [43,44]. Furthermore, it has been reported that a CNVR located on BTA18 is associated with the index of total merit and protein production, fat production and herd life in Holstein cattle [45]. These demonstrate that CNVs can be considered as promising markers for some traits or diseases in domestic animals.

Currently, there are two main platforms, i.e., comparative genomic hybridization (CGH) arrays [46-48] and SNP arrays [1,49,50], which have been extensively used in human and animals for CNV screens. The advantages and disadvantages associated with each platform were compared in [51,52]. CGH arrays have the highest signal-to-noise ratios, but give relatively low or intermediate resolution in CNV detection. SNP arrays provide high resolution of CNVs and are more convenient for high-throughput analysis and follow-up association studies due to the quantification of allele-specific copy number [51-53]. Therefore, many studies pay more attention to CNV detection based on SNP arrays, particularly along with the increasing availability of high density SNP arrays. In recent years, advances in next-generation sequencing have provided a new platform for more detailed characterization of CNVs in human and animal genomes [3-5,8,16]. But it is still too expensive for detecting CNVs in a large-scale population. In addition, methods for CNV detection using sequence data are still limited and more comprehensive algorithms or programs are needed for sequence-based CNV detection with higher resolution and sensitivity.

In the present study, we investigated genome-wide characteristics of CNVs in Chinese Hosltein cattles by using the bovine high-density (770K) SNP arrays. Consequentially, we identified 358 candidate CNV regions on 29 autosomes and 9 candidate CNV regions on the X chromosome. The result is an important complementary to the CNV map in the cattle genome, which provides an important resource for studies of genomic variation in the cattle genome.

## Results

### Genome-wide detection of CNVs

**Figure 1:**
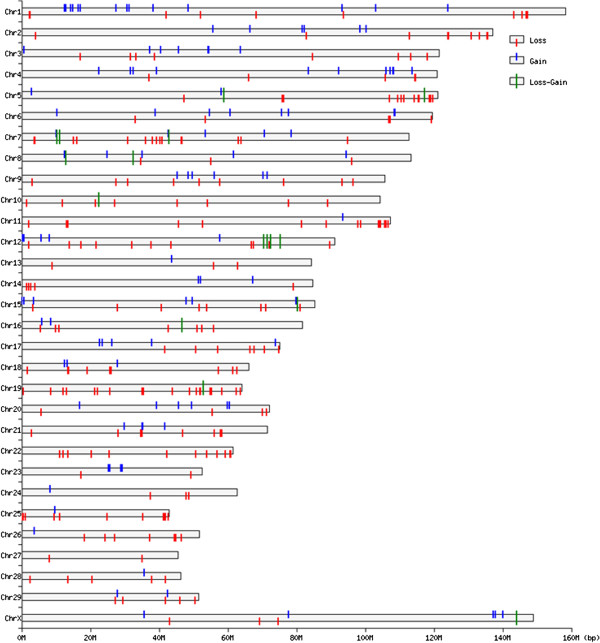
Distribution and status of detected CNVRs across the bovine genome (based on the bovine UMD3.1 assembly).

**Figure 2:**
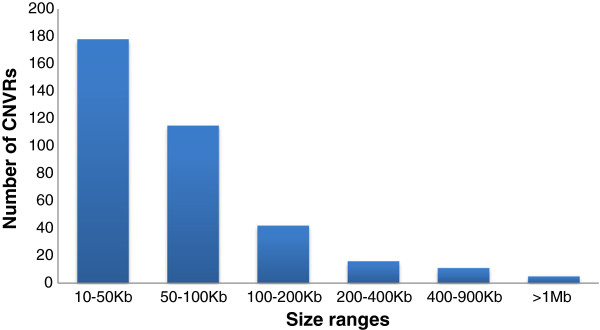
Size distribution of CNVRs detected in this study.

In total, 1733 CNVs on autosomal chromosomes and 603 on the X chromosome were detected using PennCNV. By aggregating overlapping CNVs, a total of 367 CNVRs (358 on autosomes and 9 on the X chromosome) were identified (Figure 1, Additional file 1: Table S1 and Table S2), which cover 42.74 Mb of the cattle genome and correspond to 1.61% of the genome sequence. The 358 CNVRs on autosomes cover 34.45 Mb and 1.29% of the genome sequence of autosomes, but the numbers of CNVRs on each chromosome very significantly (from 2 on BTA27 to 24 on BTA1). The lengths of them range from 10.76 Kb to 2.81 Mb with an average of 96.23 Kb and a median of 50.69 Kb.The ratio of the total CNVR length on a chromosome to the chromosome length varies from 0.22% to 6.54% (Additional file 1: Table S8). Chromosome 19 has the densest CNVRs with an average distance of 3.05Mb between CNVRs. The number of SNPs in each CNVR varies from 10 to 181. Among these CNVRs, 232, 111 and 15 of them are in loss, gain and both (loss and gain) status, respectively. The frequencies of these CNVRs in the study population range from 1.17% (one in 85) to 98.82% (84 in 85). In particular, there are 79 CNVRs with frequency >5% and 43 CNVRs > 10%, respectively. The CNVR with the highest frequency (98.82%) is on BTA 12. The detailed description of each CNVR identified on autosomes is given in Additional file 1: Table S1. The 9 CNVRs on the X chromosome cover 8.29Mb and 5.57% of the genome sequence of the X chromosome. The lengths of them range from 29.07 Kb to 4.79 Mb with a mean of 920.76 Kb and a median of 183.08 Kb. Among these CNVRs, 3 are in loss status, 5 in gain status and 1 in both status. The frequencies of these CNVRs range from 1.13 to 95.45%. Specifically, there are 5 CNVRs with frequency >15%. The detailed description of each CNVR identified on the X chromosome is given in Additional file 1: Table S2. It should be noted that the biggest CNVR, either among all CNVRs on autosomes or on the X chromosome, was detected in almost all animals. Further, out of all of the CNVRs detected, 178 (48.5%) have size less than 50 Kb (Figure 2).

### Gene content of CNVRs

A total of 610 genes within or overlapped with the CNVRs were retrieved from the Ensembl Genes 69 Database http://asia.ensembl.org/biomart/martview/, including 554 protein-coding genes, 20 pseudogenes, 14 miRNA, 12 snRNA, three snoRNA, three rRNA, two miscRNA and two retrotransposed gene (Additional file 1: Table S4). Nearly 60% (218) of the CNVRs encompass one or more annotated genes, while 40% (149) of them without any annotated genes.

After converting the bovine Ensembl gene IDs to their orthologous associated human gene IDs, we found 447 human orthologous genes (Additional file 1: Table S4), of which 374 are included in the Human Database of Genomic Variants http://dgvbeta.tcag.ca/dgv/app/home?ref=NCBI36/hg18. The GO analysis for the 447 genes show that genes of the terms of cognition, environmental response, olfactory receptor activity and neurological system process are dominantly represented in the bovine CNVRs (Additional file 1: Table S5). The KEGG pathway analysis revealed that these genes are mainly represented in the pathway of olfactory transduction (Additional file 1: Table S6).

### CNV Validation by qPCR

Quantitative PCR (qPCR) was performed to validate 17 CNVRs chosen from the CNVRs detected in the study. One or two pairs of primers were designed for each CNVR. These CNVRs represent different status of copy number variation (i.e., loss, gain and both) and different CNVR frequencies (varied from 1.17 to 98.86%) (Additional file 1: Table S3). For each CNVR, 14 positive samples (i.e., samples containing CNVR judged by PennCNV) on average were tested. For CNVRs with lower frequencies all positive sample(s) were tested, while for CNVRs with higher frequencies part of positive samples were tested. In addition, a certain number of random negative samples were also tested as negative control in qPCR.

**Figure 3:**
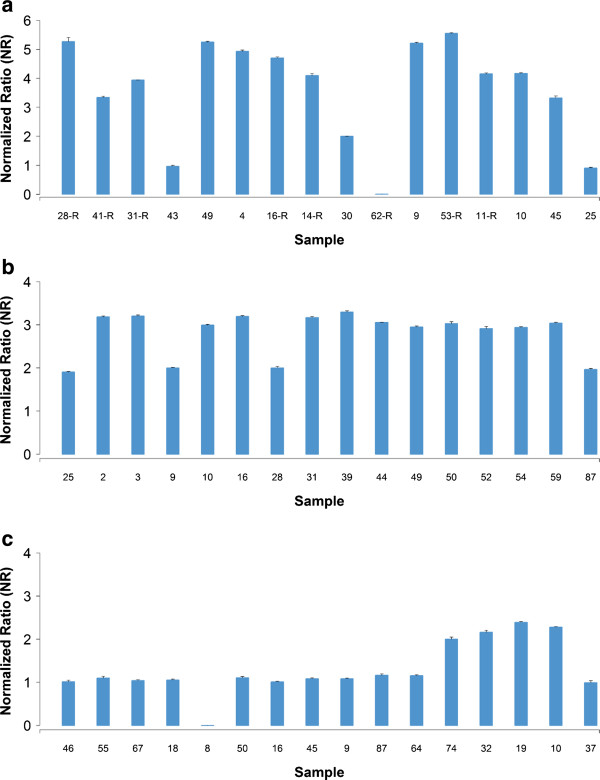
**Results of qPCR validation for three CNVRs (ID=221, 139 and 346).** Normalized ratio (NR) around 2 indicates normal status (no CNV), NR around 1 or 0 indicates one or two copies loss, and NR around 3 or above indicates one or more copies gain. The error bars represent the standard error among three technical replicates. (**a**) Results for a both type of CNVR (ID=221), (**b**) results for a gain type CNVR (ID=139) and (**c**) results for a loss type of CNVR (ID=346).

Of the 17 CNVRs, 13 (76.5%) (See Additional file 1: Table S3) were confirmed by qPCR. The average size of the 13 confirmed CNVRs and the 4 unconfirmed CNVRs were 731.76 Kb and 62.99 Kb, respectively. Additionally, the proportions of confirmed positive samples varied from 33.3% to 100% for different confirmed CNVRs. However, for some CNVRs, negative samples were also confirmed to contain CNV (false negative) with an average false negative rate of 20.1%. Figure 3a-c illustrates the qPCR results for three confirmed CNVRs of different types (loss, gain and both).

## Discussion

Several algorithms for CNV detection based on SNP array have been developed and implemented in different programs, e.g., PennCNV, GADA, cnvPartition, etc. Each algorithm has its strengths and weaknesses as summarized by Winchester et al. [54]. However, most CNV studies based on SNP array in human and animals used only the PennCNV software (e.g., Bae et al. [23]; Hou et al. [55]; Glessner et al. [56]), although Winchester et al. [54] recommended using multiple algorithms on a single dataset to produce the most informative and reliable results. In this study, we chose to use only PennCNV for two reasons: 1) some studies indicated that PennCNV is more reliable in detecting CNVs than some other algorithms (e.g., Marenne et al. [57]); 2) When using multiple algorithms, it is difficult to made a conclusion how many CNVs there should be, if we only accept the CNVs commonly detected by all algorithms, there are certainly a lot of CNVs being missed, on the other hand, if we accept all CNVs detected by different algorithms, there must be a lot of false positive CNVs. Furthermore, in this study, CNV was inferred with a strict criterion (i.e., it must contain ten or more consecutive SNPs) to reduce the risk of high false positive rate due to use only one algorithm.

So far, CNV detection in the cattle genome has been reported in several studies using different technological platforms, i.e., comparative genomic hybridization (CGH) array [24,25], bovine 54 K SNP Beadchip [23,26,58], bovine HD SNP Beadchip [55] and next- generation sequencing [4,5,8]. In the present study, using the bovine HD SNP Beadchip, we identified 358 autosomal and 9 X-chromosomal CNVRs. We assessed our results by comparing with previous published cattle CNVs. Since most existing cattle CNVs are mapped on the Btau_4.0 genome builds, we converted our results from UMD3.1 to BTAU_4.0 using the UCSC liftOver tool [59]. 278 out of 367 CNVRS (277 on autosomes and 1 on the X chromosome) were successfully converted from UMD 3.1 to BTAU_4.0 genome assembly (Additional file 1: Table S9). Here, we only compare the CNVRs on autosomes, since the X chromosome was excluded in most of other CNV studies. The comparison results are presented in Table 1.

**Table 1:** Comparison between results of the current study and results from other studies

	**Findings from different studies**					**Overlapped CNVRs of this study**			
	**Study**	**Breed**	**Sample**	**Count**	**Total length (Mb)**	**Count**	**Percentage of count**	**Total length (Mb)**	**Percentage of length**
CGH-based Studies	Fadista et al. [25]^a^	4	20	266	16.6	27	9.7%	1.71	8.7%
	Liu et al. [24]^b^	17	90	177	28.1	16	5.8%	1.58	8.1%
SNP-based Studies	Hou et al. [26]	21	521	682	139.8	55	19.8%	5.35	27.3%
	Bae et al. [23]	1	265	368	63.1	35	12.6%	2.00	10.2%
	Hou et al. [58]	1	472	811	141.8	51	18.3%	3.41	17.4%
	Jiang et al. [60]	1	2047	101	23.8	14	5.0%	2.50	12.8%
	Hou et al. [55]	27	674	3346	142.7	119	42.8%	7.59	38.7%
Resequencing-based Studies	Bickhart et al. [8]	3	5	1265	55.6	19	6.8%	0.855	4.4%
	Zhan et al. [5]	1	1	520	3.6	13	4.7%	0.253	1.3%
	Stothard et al. [4]	2	2	790	3.3	14	5.0%	0.380	1.9%
This study				278	19.6				

Based on Btau4.0 bovine genome assembly.

a: CNVRs on Chr Un and mitochondrial sequence are excluded;

b: CNVRs on Chr Un are excluded.

It is notable that only a small proportion of CNVRs in our study overlapped with other studies. Similar situation were also reported in human and other mammal CNV studies. The inconsistence between results of different studies can be due to the differences in size and structure of the study population, platform and algorithm for CNV calling, and CNV (CNVR) definition between these studies as well as potential technical and random errors. It also suggests that a vast amount of CNVs existing in the cattle genome has not been discovered. We summarized the detailed characteristics of the cattle CNVRs on autosomes reported in all studies (Table 2). In general, the CNVRs identified based on the 54K SNP chip are much longer than those based on CGH array, HD SNP chip or sequence data, while the CNVRs based on sequence data are the shortest. It can be explained that the illumina 54 K SNP panel with an average gap size of 53 Kb is not sufficient to detect small CNVs in cattle. In the present study, we performed CNV detection using the BovineHD beadChip with an average gap size of 3.43 Kb. It should recognize that a CNV was defined to contain 3 or more consecutive SNPs in all of the previous CNV studies based on the 54 K SNP chip. Although we defined a CNV to contain ten or more consecutive SNPs in the present study, the identified CNVRs are much shorter on average than those based on the 54 K SNP chip. Particularly, in comparison between this study and our previous study using the 54K SNP chip [60], both of which performed CNV detection in Chinese Holstein cattle, besides the difference in CNVR size, much more CNVRs were identified in this study, although the population used in this study is much smaller than that in the previous study. This demonstrates that the HD SNP chip provides an advantage over the 54 K SNP chip to detect CNV since it can detect many small CNVs in addition to the large ones. This was also proved by the study of Hou et al. [26,55], who used the HD SNP array and identified much more CNVRs than their previous study based on the 54K SNP array (3,346 vs 682). In comparison with the study of Hou et al. [55], much fewer CNVRs were identified in the present study, although both studies are based on the HD SNP array. This should be explained that a much larger study population with multiple breeds (674 animals of 27 breeds) was involved and a less strict creterion was applied to define CNV (at least three consecutive SNPs) in their study. If we apply the same criterion to define CNV, 792 CNVRs could be obtained.

**Table 2:** Characterization of cattle CNVRs on autosomes based on different platforms

**Study**		**Summary statistics of CNVRs**						
		**Mean(Kb)**	**Median(Kb)**	**Min(Kb)**	**Max(Kb)**	**Standard deviation**	**Total length(Mb)**	**No. of CNVR**
CGH-based Studies	Fadista et al. [25]	62.05	9.73	1.72	2031.34	155.05	15.76	254
	Liu et al. [24]	153.75	86.19	18	1261.9	178.29	25.06	163
	averge	107.90	47.96	9.86	1646.62	166.67	20.41	208.50
SNP-based Studies (54k chip)	Hou et al. [26]	204.97	131.18	32.57	5569.1	296.49	139.79	682
	Bae et al. [23]	171.49	128.33	25.35	967.18	135.67	63.11	368
	Hou et al. [58]	174.88	128.27	25.8	1417.77	157.98	141.83	811
	Jiang et al. [60]	235.46	156.54	27	1312.35	225.47	23.78	101
	averge	196.70	136.08	27.68	2316.60	203.90	92.13	490.50
SNP-based Studies (HD chip)	This study*	96.23	50.64	10.76	2806.42	201.99	34.45	358
	Hou et al. [55]*	42.73	15.65	1.03	4345.96	148.5	146.91	3438
	averge	69.48	33.15	5.90	3576.19	175.25	90.68	1898
Resequencing-based Studies	Bickhart et al. [8]	42.89	22.76	10.02	510.94	54.65	47.99	1119
	Zhan et al. [5]	6.98	3.8	3.17	129.97	10.29	3.63	520
	Stothard et al. [4]	4.16	3.17	1.84	28.03	2.96	3.29	790
	averge	18.01	9.91	5.01	222.98	22.63	18.30	809.67

*: Based on UMD3.1 bovine genome assembly, others based on Btau4.0 bovine genome assembly.

In order to confirm these potential CNVRs, we performed quantitative PCR for 17 randomly selected CNVRs and 13 of them (76.5%) were confirmed successfully. The percentage is higher than the results of previous reports in animals [6,21,26]. It can be explained that the high density probe of the BovineHD beadChip and the strict CNV definition (i.e., it must contain ten or more consecutive SNPs) were used in this study. Most of the positive samples revealed by PennCNV prediction agreed well with the qPCR experiments. However, there are also a small proportion of false negative samples. The average false negative rate for each CNVR was 20.1%. False negative identification in CNV detection has also been reported in previous studies [6,21,22,61]. It demonstrates that although the strict criteria of our study can minimize the false-positive rate, it also simultaneously resulted in false-negative rate. Besides, some positive samples which are not confirmed may not be really the false positive ones. Because the primers used to confirm the CNVRs may have been designed outside the actual boundaries for some individuals as the CNVRs are the union of CNVs in different animals.

The CNVRs identified in our study cover or overlap with a total of 610 genes, of which 447 are orthologous with corresponding human genes. Most (374) of these orthologous genes are included in the Human Database of Genomic Variants (Additional file 1: Table S4), i.e. they are also related with CNVs in human. Especially, the functions of some genes are enriched in the same GO terms (such as plasma membrane, cognition and sensory perception) and pathways (such as olfactory transduction) as those reported in other CNV studies in cattle and other mammals [1,17,25,26,55]. We also compared the 367 CNVRs identified in this study with the reported QTL collected in the cattle QTL database (http://www.animalgenome.org/QTLdb/doc/genome_versions#UMD_3.1). Since some QTL have too large confidence interval, we focused on QTL with confidence interval less than 30cM and considered those QTLs with overlapped confidence intervals greater than 50% as the same QTL. In this way, we identified 259 QTL in total. 341 out of the 367 CNVRs harbor or partially overlap with 182 (70%) QTL (Additional file 1: Table S7). These QTL are involved in many traits, such as milk production traits, carcass traits, reproduction traits, and health traits (see Additional file 1: Table S7).

## Conclusions

In summary, we identified 367 CNVRs distributed on all of the 29 autosomes and the X chromosome of the bovine genome using the BovineHD beadChip. qPCR was performed for 17 CNVRs to validate the results and 13 (76.5%) of them were confirmed successfully. Six hundred and ten genes are covered by or overlapped with these CNVRs, most of which are also reported to be related with CNVs in the human genome. Compared with the results of CNV studies based on bovine 54K SNP array, CNVs detected in this study have smaller mean size, higher resolution and higher qPCR validation rate, suggesting that CNV detection based on high-density SNP arrays can significantly improve the accuracy and sensitivity of CNV calling. Findings in our study enhance the CNV map in the cattle genome and provide meaningful information for investigation of associations between CNVs and important traits in cattle in further study.

## Methods

### Sample collection and genotyping

The study population consisted of 96 Chinese Holstein cattle with unknown relationship among them, including 86 bulls and 10 cows. The Chinese Holstein originated from crosses of European Holstein-Friesian with Chinese Yellow cattle about 70 yr ago. Since then, continuous introgression of foreign Holstein genes (live bulls, semen, and embryos), mainly from North America, have been conducted. Therefore, the current population has a close relationship with the North American Holstein.

Genomic DNA samples were extracted from blood samples of cows and semen samples of bulls. The blood samples were collected along with the regular quarantine inspection of the farms. The concentration and the purity of genomic DNA were assessed on the Nanovue Spectrophotometer. All samples were genotyped with the Illumina High-Density BovineSNP beadChip containing 777,692 SNPs that uniformly span the bovine genome with an average gap size of 3.43 Kb and a median gap size of 2.68 Kb. All the markers were clustered and genotyped using the BEADSTUDIO software (Illumina). The whole procedure for collection of the blood samples was carried out in strict accordance with the protocol approved by the Animal Welfare Committee of China Agricultural University (Permit number: DK996).

In order to increase the confidence in CNV detection, strict quality control of the genotype data was applied according to the signal-to-noise ratios of each sample. The quality of the final data sets was assessed by the standard deviation of Log R ratio (LRR_SD) and B allele frequency drift (BAF_drift) of each sample. Only those samples with LRR_SD <0.30 and BAF drift <0.01 were included. Finally, 85 (10 cows and 75 bulls) with average call rate of 99.9% out of the 96 samples were remained for CNV detection on autosomal chromosomes and 88 (10 cows and 78 bulls) for CNV detection on the X chromosome.

### Identification of cattle CNVs

The PennCNV software [62] was employed to infer cattle CNVs in this study. This algorithm incorporates multiple information, including total signal intensity (LRR) and allelic intensity ratio (BAF), the population frequency of B allele (PFB) of SNPs, the distance between neighboring SNPs and the pedigree information where available. The LRR and BAF of all SNPs for all samples were exported from the BeadStudio software (Illumina). The PFB file was generated based on the BAF of each SNP. The SNP genomic positions on chromosomes were derived from the bovine UMD3.1 genome sequence assembly [63]. Furthermore, the signal intensity of each SNP which is subject to genomic waves was adjusted for the GC content of the 500Kb genomic region of its both sides using the *-gcmodel* option of PennCNV. PennCNV was run using the *–test* option without considering pedigree information since the relationship of the individuals in our study population is unknown. The analysis of the X chromosome and autosomes were separately performed in this study.

Following the CNV studies using high density SNP chip in human [64,65], we define a CNV as it must contain ten or more consecutive SNPs and a CNV region (CNVR) as the overlapping region covered by the CNVs identified across all samples according to Redon et al. [1].

### Gene contents and functional annotation

Gene contents of the identified CNVRs were retrieved from the Ensembl Genes 69 Database using the BioMart Database http://asia.ensembl.org/biomart/martview/ based on the bovine UMD3.1 sequence assembly. Gene Ontology (GO) analysis [66] and .Kyoto Encyclopedia of Genes and Genomes (KEGG) pathway analyses [67] were performed for genes that were completely included within or overlapped with the CNVRs with the DAVID bioinformatics resource [68] [http://david.abcc.ncifcrf.gov/summary.jsp] to determine their functional enrichment. Before these analyses, these bovine Ensembl gene IDs were converted to their human ortholog Ensembl gene IDs with BioMart since the annotated genes in the cattle genome are limited. We also compared these human ortholog genes with the CNV related genes reported in the Human Database of Genomic Variants (DGV) http://dgvbeta.tcag.ca/dgv/app/home?ref=NCBI36/hg18.

### qPCR validation

Quantitative real time PCR (qPCR) was used to validate CNVRs detected in the study. The relative comparative threshold cycle (2^-≥≥C^T) method was used to quantify copy number changes by comparing the ΔCt [cycle threshold (Ct) of target region minus Ct of control region] value of samples to be tested to the ΔCt of a calibrator without CNV [69,70]. CNVRs were tested by using SYBR Green chemistry as recommended by the manufacturers. We designed the PCR primers using the Primer 3 webtool (http://frodo.wi.mit.edu/primer3/). For each target CNVR, two pairs of primers were designed considering the uncertainty of the CNVR boundaries. Moreover, the In-Silico PCR program from the UCSC browser (http://genome.ucsc.edu/) was used for *in silico* specificity analysis to ensure the primers only matching the sequence of interest. A serial diluted genomic DNA samples from a common cattle was used as template for creating a standard curve of each primer. Amplification efficiencies of all primers were calculated based on the standard curves. The copy number of each CNVR was compared with a region in the control gene *Basic transcription factor* 3 (*BTF3*) as done in previous studies [23]. All PCR primers were designed based on its reference sequence in NCBI. PCR amplifications were performed in a total volume of 20 μL consisting of the following reagents: 1 μL DNA (around 50 ng), 1 μL (20 pM/μL) of both forward primer and reverse primer, 10μL of Master Mix (2×) and water (Roche Applied Science). All RT-PCRs were run in triplicate. PCRs were run as follows: 5min at 95°C followed by 40 cycles at 95°C for 10 sec and 60°C for 10 sec. All PCRs were performed in 96-well clear reaction plates (Roche Applied Science). The average C_T_ value of three replications of each sample was calculated and normalized against the control gene with the assumption that there are two copies of DNA segment in the control region. For each CNVR to be validated, a value from the formula 2×2^-≥≥C^T was calculated for each individual. For autosomal chromosomes, a value around 2 indicates the individual is in normal status (without CNV), a value around 3 or above indicates it is in gain status, and a value 1 or below indicates it is in loss status. For X chromosome, the judgment for cows is the same as stated above. For bulls, the corresponding values for normal, loss and gain status were around 1, 0 and 2 or above, respectively.

## Competing interests

The authors declare that they have no competing interests.

## Authors’ contributions

JL carried out the experimental validations and wrote the manuscript. JJ carried out computational analysis. YJ participated in the statistical analysis of qPCR. ZQ conceived of the study and led in its design and helped to draft the manuscript. LX, WJ, WH, DX and LJ contributed to the sample genotyping, data analysis and interpretation of data. All authors read and approved the final manuscript.

## Supplementary Material

Additional file 1: Table S1 The detailed features of CNVRs on autosomes identified in this study. **Table S2.** The detailed features of CNVRs on on the X chromosome identified in this study. **Table S3.** Information of the 17 CNVRs to be validated by qPCR and the primers used for qPCR. **Table S4.** Genes covered by or overlapped with CNVRs, their orthologs in human genome and comparison with genes included in Human Database of Genomic Variants (DGV). **Table S5.** Ontology (GO) analyses of genes in CNVRs detected in this study. **Table S6.** Pathway analyses of genes in CNVRs detected in this study. **Table S7.** QTLs harbored within or overlapped with identified CNVRs across the bovine genome. **Table S8.** Ratios of total CNVR length on a chromosome to the chromosomal length. **Table S9.** Genome coordinates of CNVRs converted in BTAU4.0 bovine genome assembly. Click here for file
